# Supplementary material for: Geographical drivers and climate-linked dynamics of Lassa fever in Nigeria
Source: Nat Commun. 2021 Oct 1;12:5759. doi: 10.1038/s41467-021-25910-y (PMC8486829; doi:10.1038/s41467-021-25910-y)
Supplement: Supplementary file 1 — Supplementary Information [file 41467_2021_25910_MOESM1_ESM.pdf]

Supplementary Information for Redding *et al.* – Geographical drivers and climate-linked dynamics of Lassa fever in Nigeria

Supplementary Figure 1: Lassa fever case time series from Nigeria Centre for Disease Control reporting regimes. Graphs summarise weekly surveillance data aggregated across all local government authorities (LGAs), from January 2012 to December 2019, and show the differences between two surveillance regimes. Bar plots show monthly total cases from the long-term Weekly Epidemiological Reports (W.E.R., top) and more recent Situation Reports (SitRep, bottom) regime, with bar heights representing the total LF cases from all epidemiological weeks starting during a given month, split into suspected (grey) and confirmed (black) cases. Weekly case accumulation curves per-surveillance regime, per-year show total reported cases (including both suspected and confirmed; top graphs) and confirmed only (bottom graphs). LF trends during the overlap period between the two regimes are similar (January 2017 to March 2018) but the SitRep data (based on the most current reporting regime and including post-hoc follow-ups to ensure accurate counts) more clearly show the very large increase in both suspected and confirmed case reports in 2018. The full time series used in analyses (Figure 1) includes W.E.R. data from 2012 to 2016, and SitRep data from 2017 to 2019.

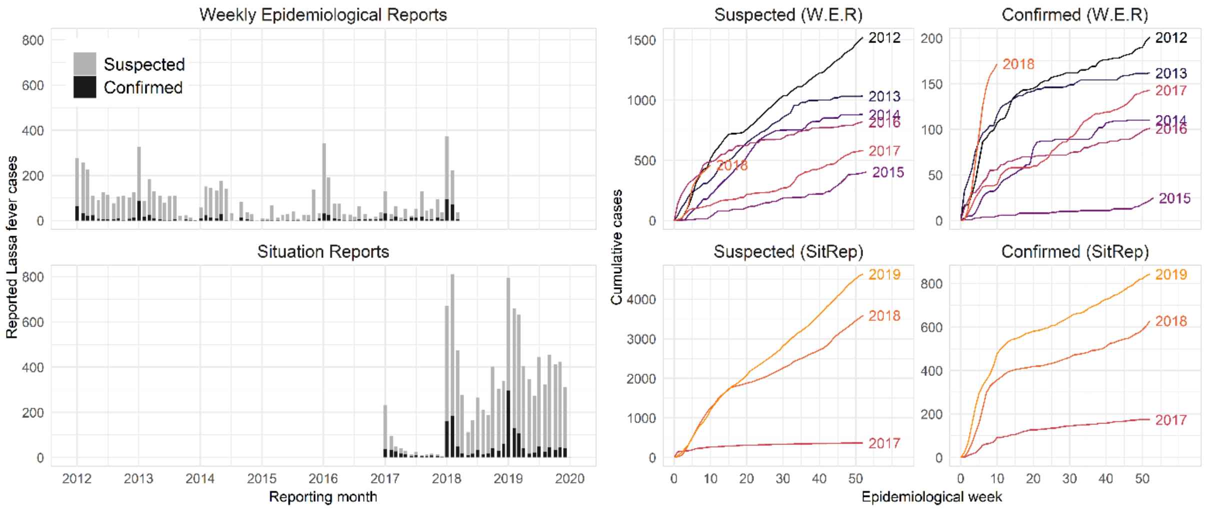

Supplementary Figure 2: Annual spatiotemporal random effects from models of Lassa fever occurrence and incidence across Nigeria. Models of LF occurrence and incidence (2016-2019) included year-specific, spatially-structured (conditional autoregressive; *u*) and unstructured (*v*) random effects at LGA level (jointly specified as a Besag-York-Mollie model). These account for both spatial autocorrelation in environmental and reporting processes, and ongoing expansion of surveillance throughout the reporting period. Dark blue maps show the annual fitted LGA-level random effect (i.e. $u + v$; see Methods) for the full models with socio-environmental covariates, for occurrence (A; colour denotes log odds of occurrence) and incidence (B; colour denotes log incidence). Diverging colour maps show the difference in the fitted spatio-temporal effect between baseline (random effects only) and full models (with covariates), for occurrence (B) and incidence (D). More negative values (brown) indicate areas where including socio-environmental covariates has provided additional explanatory power (i.e. where random effects have reduced towards zero), and vice versa.

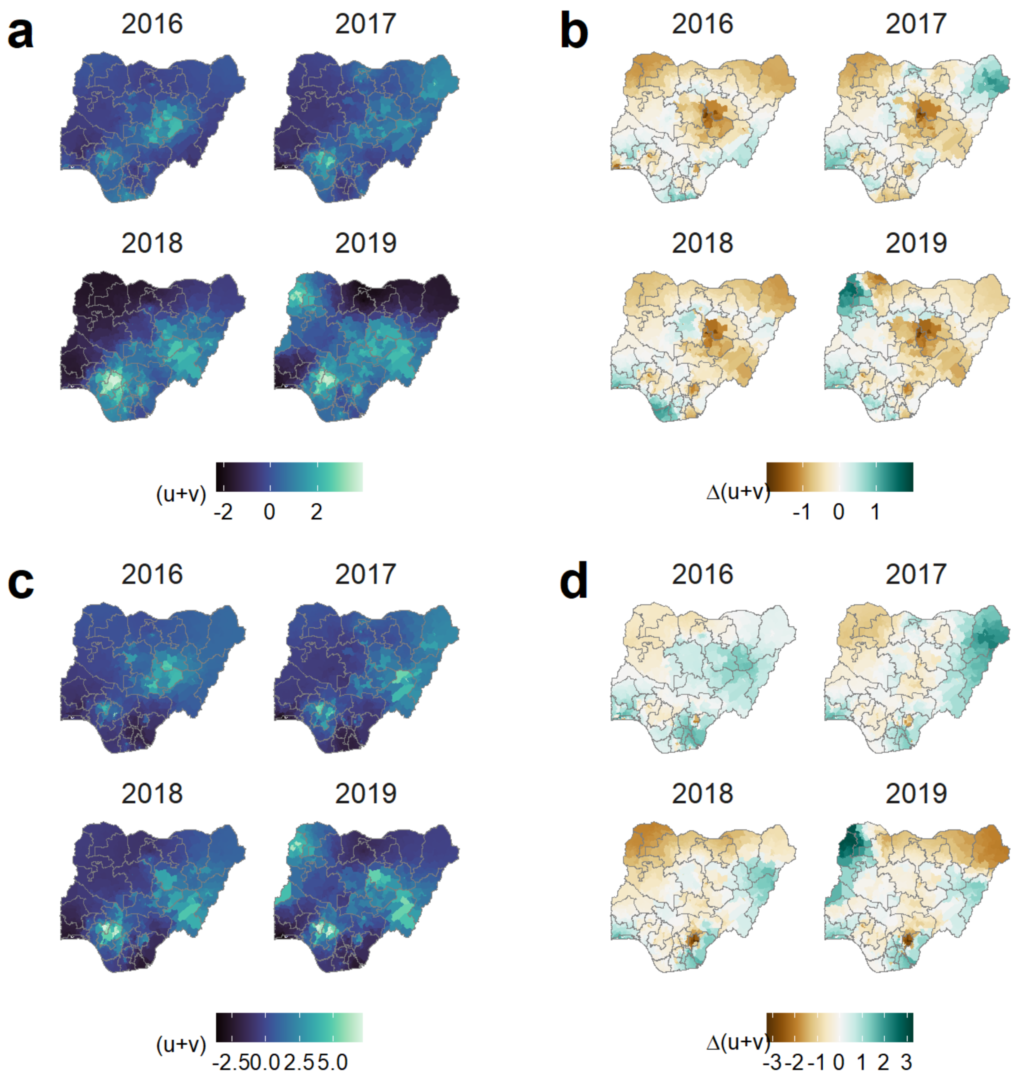

Supplementary Figure 3: Geographical cross-validation for spatial models of Lassa fever occurrence and incidence. The direction and magnitude of linear fixed effects and nonlinear climate effects in both occurrence (A-B) and incidence models (C-D) were robust to geographically-structured cross-validation (n=3096; i.e. 774 LGAs over 4 years). This involved in turn excluding all LGAs from each of 12 Lassa-endemic and non-endemic states, with point or line colour denoting the state that was excluded in each model iteration. Points and error bars show linear fixed effects (mean and 95% credible interval), and lines show fitted nonlinear effects of mean annual precipitation on either occurrence risk (odds ratio) or incidence (relative risk) (lines show posterior mean and transparent grey shading denotes 95% credible interval for each submodel). These results indicate that the findings were not overly influenced by data from any one locality.

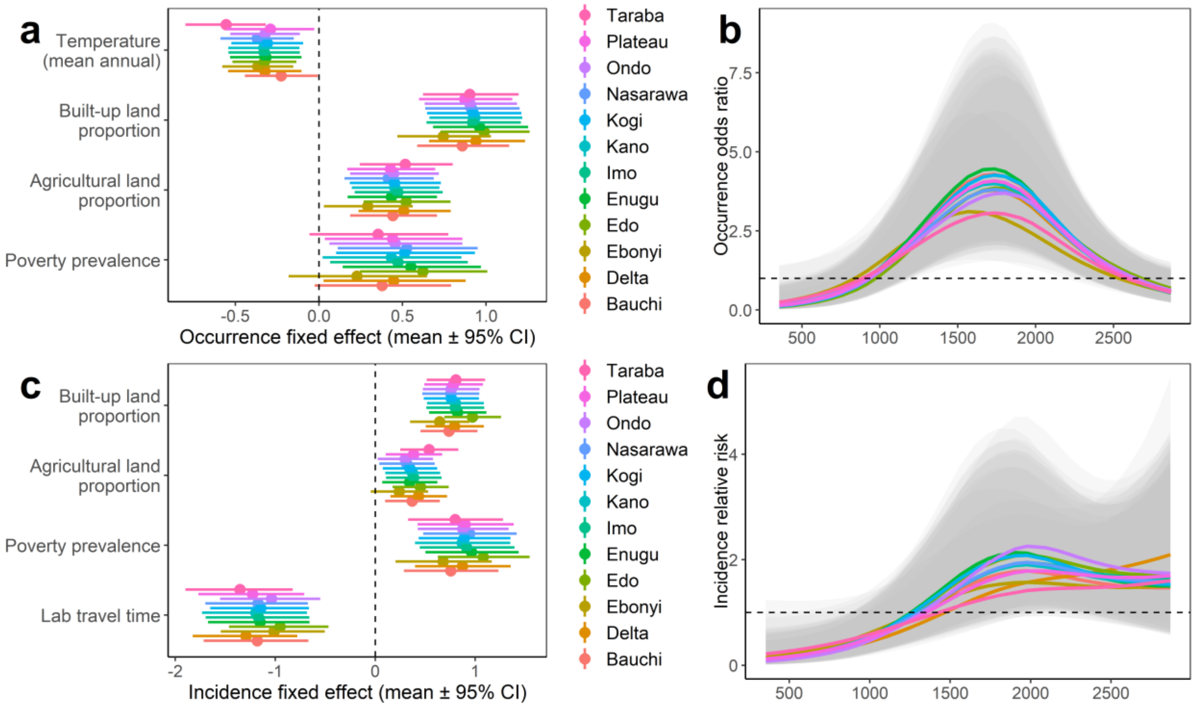

Supplementary Figure 4: Fitted spatial models of Lassa fever occurrence and incidence for spatially-aggregated districts. To examine the effects of scale on inferences, we repeated all spatial modelling after aggregating LGAs into 130 composite districts, subject to the constraints of state boundaries, producing a more even area distribution (median 6826km^2, mean 6998km^2, range 1641 – 14677km^2) (n=520, i.e. 130 districts over 4 years). The figure shows the same results as for Figure 3 (main text) at lower spatial resolution: geographical patterns of fitted occurrence (A) and incidence (B), linear fixed effects estimates (C; log odds scale for occurrence, and log scale for incidence; points and error bars denote posterior mean and 95% credible interval) and nonlinear fitted functions of total annual precipitation on occurrence (D) and incidence (E) (lines and shading denote posterior mean and 95% credible interval). All linear covariates were centred and scaled prior to model fitting, so fixed effects are comparable between covariates (i.e. each measure the effect of an increase of 1 standard deviation of the covariate on the response). Parameter estimates are provided in Supp. Table 2.

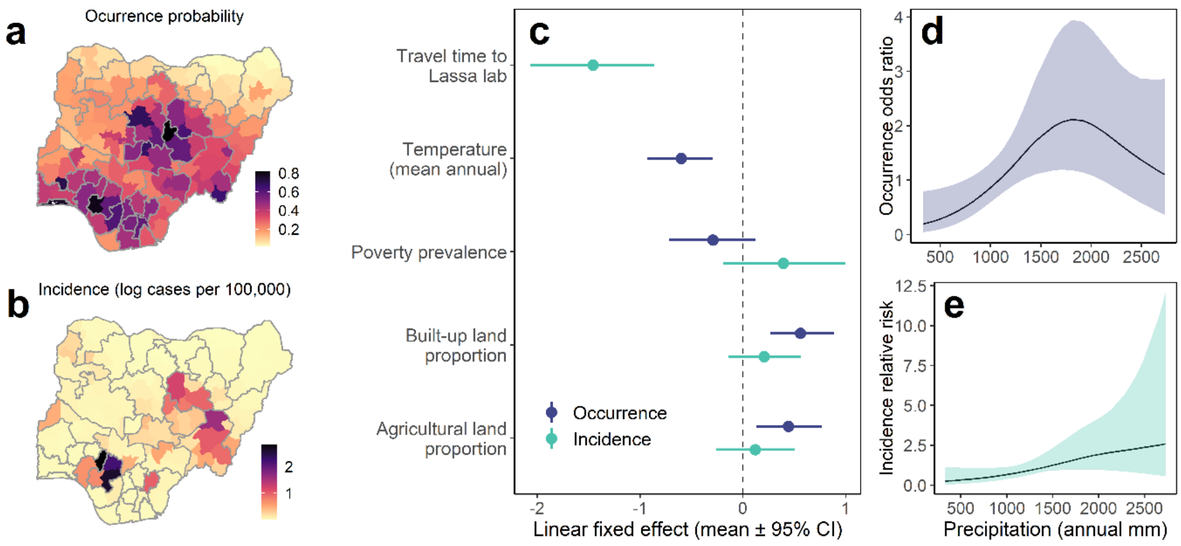

Supplementary Figure 5: Spatial projection of environmental suitability for Lassa fever occurrence and incidence across Nigeria. Maps show the contributions of climatic and socio-ecological fixed effects to the linear predictor for LF occurrence (top row; log odds scale) and incidence (bottom row; log scale). Projecting combined socioeconomic and environmental effects (left column) or environmental effects alone (climate and agriculture; right column) shows that the broad envelope of LF suitability covers much of Nigeria. However, the heterogeneous observed distribution and high-incidence hotspots in south Nigeria (Edo and Ondo states) are mainly explained by random effects (Supplementary Information Figures 2a and 2c).

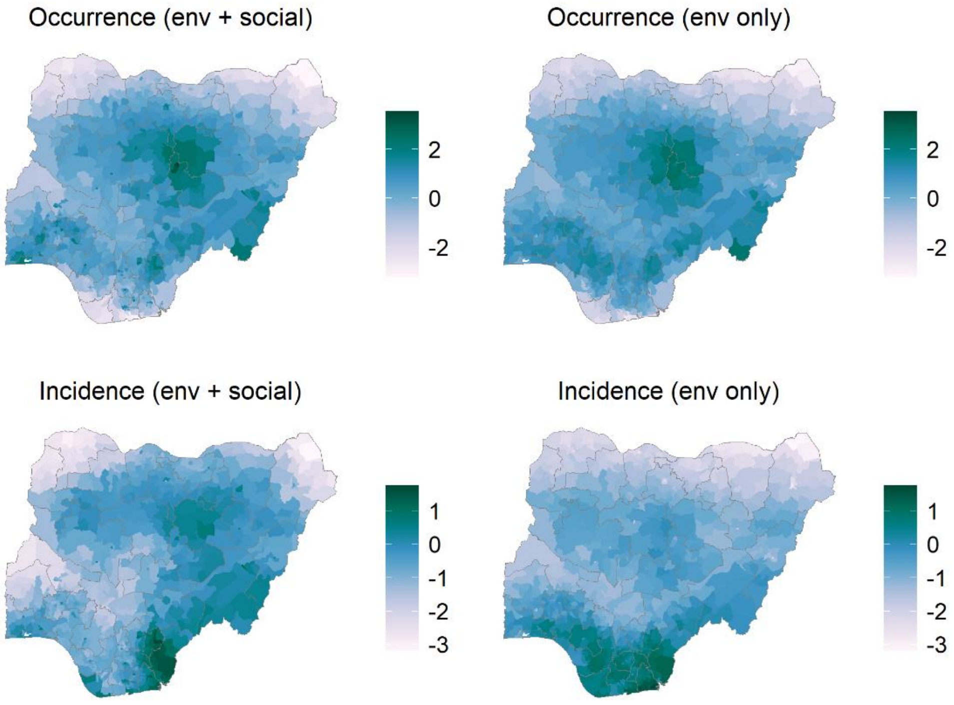

Supplementary Figure 6: Seasonal and interannual climate and vegetation dynamics in Lassa-endemic regions of south and north Nigeria. Graphs show, for south (left column) and north (right column) Lassa-endemic areas, state-level weekly mean environmental (temperature, precipitation) and vegetation values across a 60-day window prior to reporting week (i.e. at time of transmission occurring). Separate lines show trends for separate states: Bauchi, Plateau and Taraba (north) and Edo, Ondo and Ebonyi (south). Temperature estimates are daily mean (Tmean; blue), minimum (Tmin) and maximum (Tmax), derived from Climate Prediction Centre interpolated air temperature layers from NOAA. Vegetation estimates are daily mean Enhanced Vegetation Index (EVI), derived from 16-day interval EVI rasters from NASA. EVI values below a ~0.2 threshold (dotted line) indicate a lack of dense green vegetation. Precipitation estimates are daily mean rainfall, derived from daily rainfall layers from CHIRPS Africa. SPI3 shows Standardised Precipitation Index: values below 0 (dotted line) reflect drought conditions, and values above 0 reflect wetter conditions, relative to historical observed trends (1981-2020) for the same period of the year.

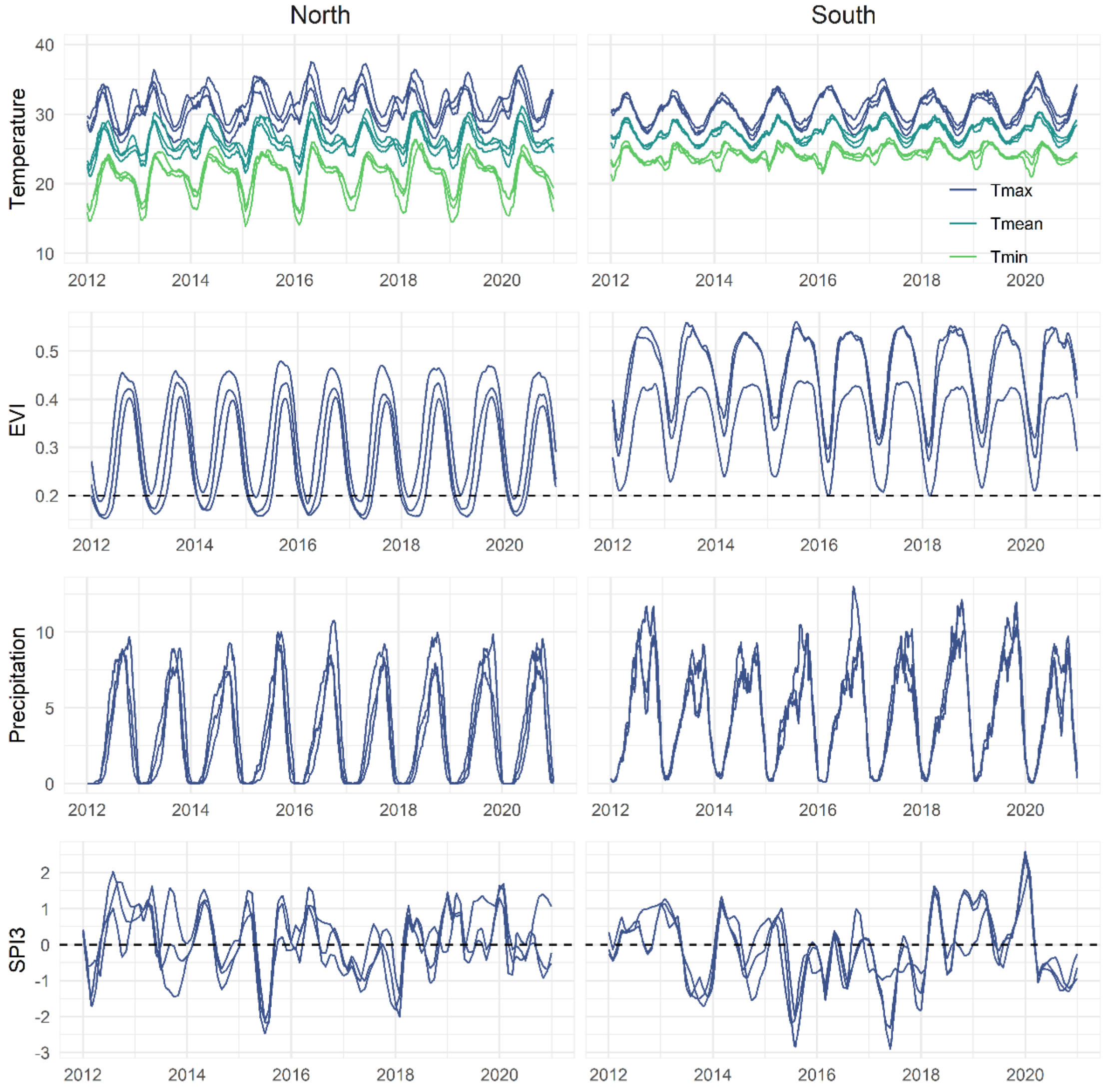

Supplementary Figure 7: Marginal effects of environment, seasonality and year on temporal Lassa fever incidence. Figures show the relative risk associated with climate and seasonality (A) and reporting and random effects (B) in south and northern states (represented in models by region-specific effects of year and season). Separate lines are exponentiated linear combinations of climate and random effects for each state, and reflect relative LF risk associated with these model components. The left column shows the combination of climatic covariates and seasonal effect (random walk of epidemiological week; see Methods), showing the expected interannual differences in LF risk associated with climate (A). The right column shows the combined effect of state-level, year and travel time to laboratory effects (i.e. random and observation-based) on relative risk, showing the rapid increase in surveillance effort associated with the 2017-19 period.

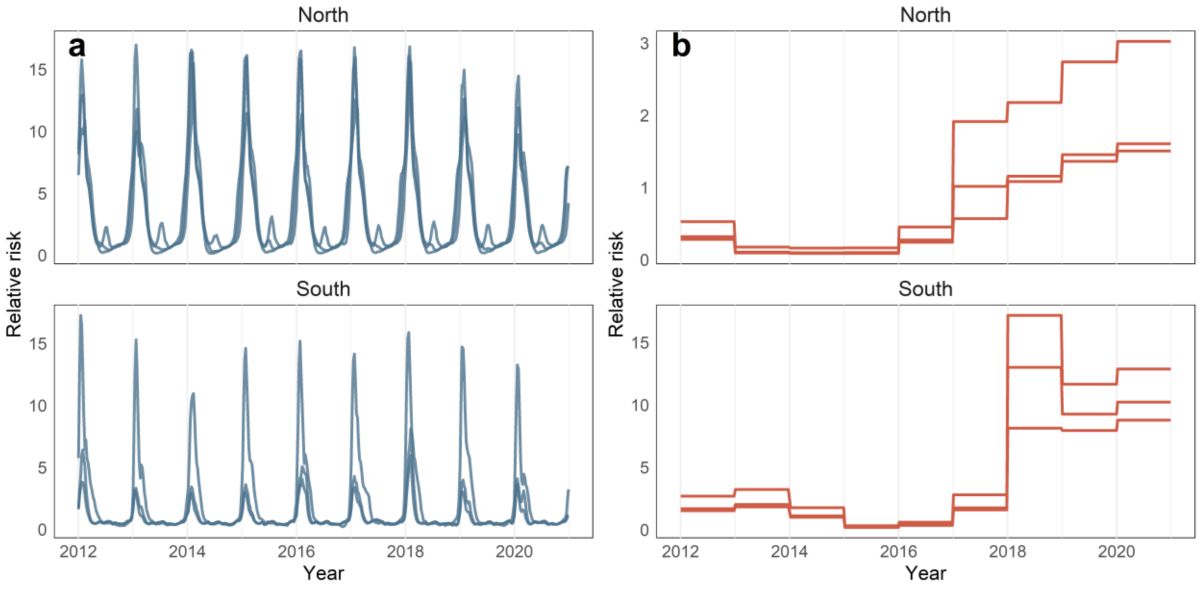

Supplementary Figure 8: Retrospective out-of-sample predictions for temporal models of Lassa fever incidence. Trend graphs show, for each state, the weekly observed confirmed LF cases (grey bars), and out-of-sample (OOS) predictions from the final climate-driven model. Red line shows the posterior median and shaded areas show the OOS 95% simulated posterior predictive interval (see Methods), calculated using 2500 samples drawn from the joint posterior. Across the entire surveillance period, the climate-driven model reduced predictive error relative to a baseline model (Table 1). Calibration was good overall although predictions were generally slightly overdispersed relative to observed cases, for both southern (90% of observations falling within 95% predictive interval; 79% falling within the 67% predictive interval) and northern states (97% of observations within 95% interval; 91% within 67% interval).

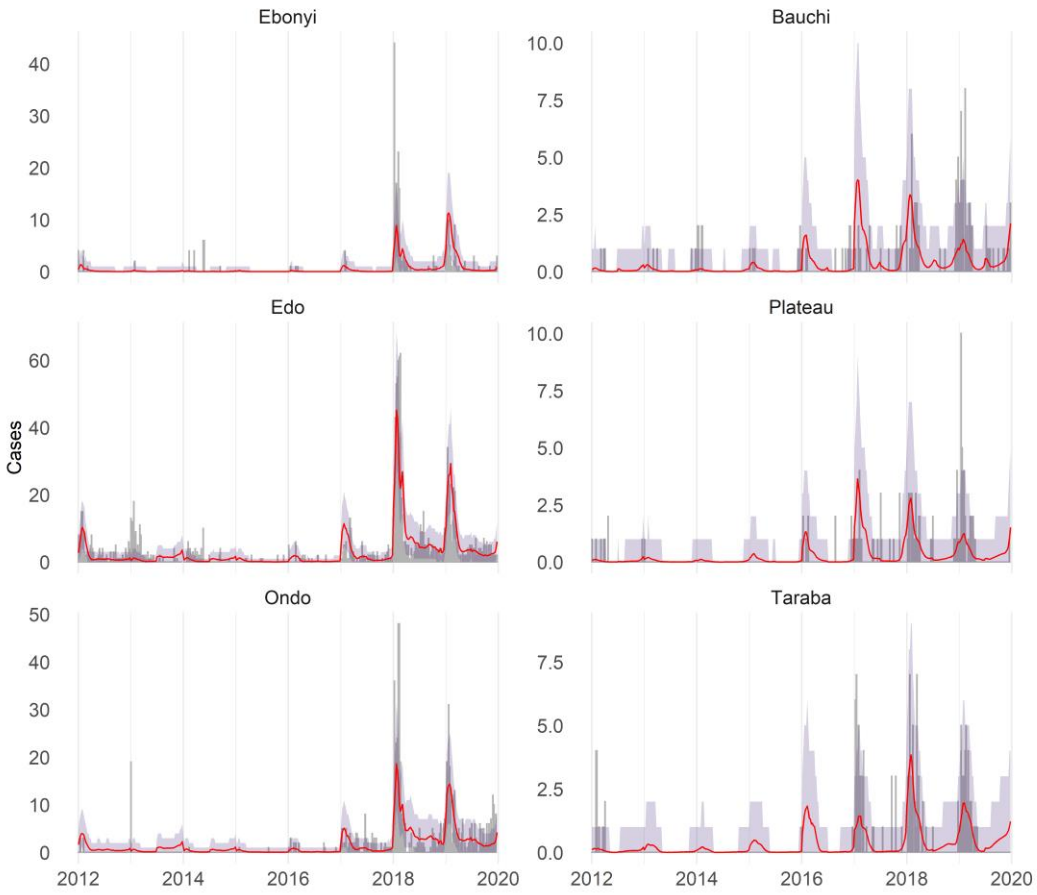

Supplementary Figure 9: Prospective predictions of Lassa fever case incidence for 2020. Graphs show, for each state, the comparison of observed weekly case counts in 2020 (grey bars) and predicted cases from baseline and climate-driven models. Lines show the posterior median predicted value for baseline (pink) and climate-driven (blue) models, and shaded area shows the 95% posterior predictive interval from the climate-driven model, both calculated using 2500 samples drawn from the joint posterior. Predictions hold all random effects at 2019 levels (i.e. assume the same effect of year). Both models substantially underpredict total cases observed (see Results), potentially because neither captures ongoing improvements in surveillance sensitivity.

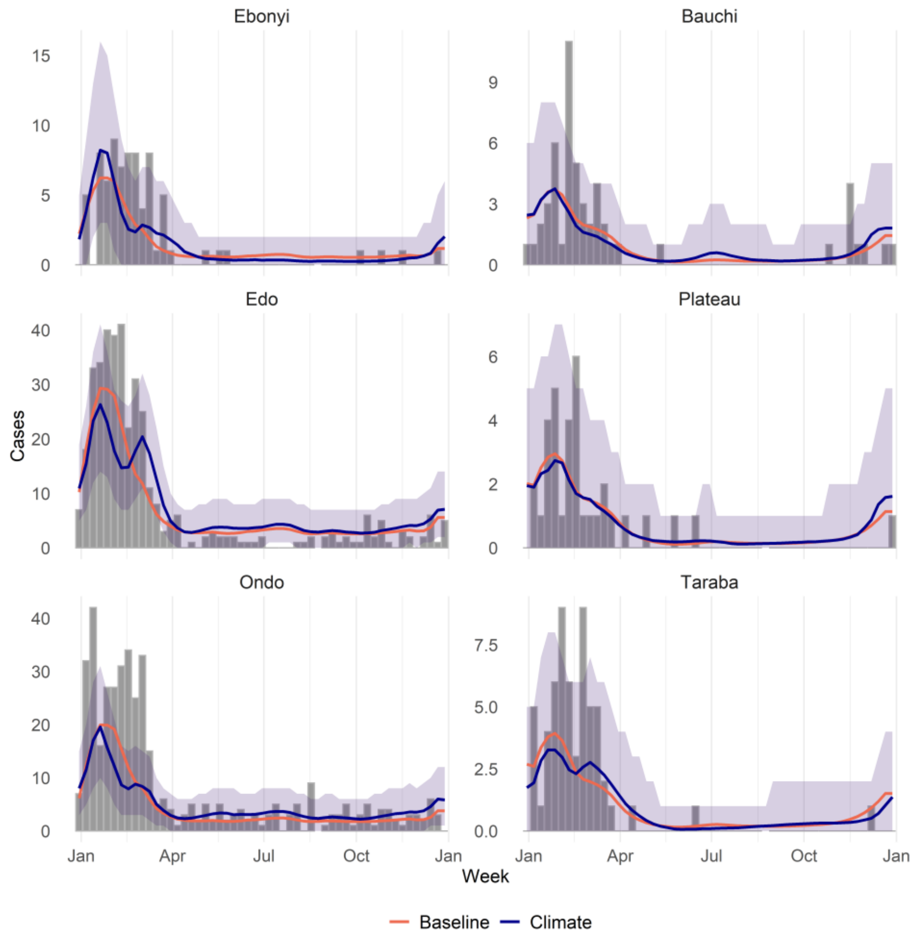

Supplementary Table 1: Clinical definitions used for diagnosis of suspected and confirmed Lassa fever cases. Clinical definitions and criteria are listed in weekly Nigeria Centre for Disease Control Lassa fever Situation Reports[1].

Term	Primary Criterion	Secondary Criterion
Alert case	Any person who has an unexplained fever (i.e. Malaria and other likely causes of fever have been ruled out), with or without bleeding<br>OR<br>Any person who died after an unexplained severe illness with fever and bleeding	
Suspected case	An illness of gradual onset with one or more of the following: malaise, fever, headache, sore throat, cough, nausea, vomiting, diarrhoea, myalgia (muscle pain), central chest pain or retrosternal pain, hearing loss and either:	a. History of contact with excreta or urine of rodents<br>OR<br>b. History of contact with a probable or confirmed Lassa fever case within a period of 21 days of onset of symptoms<br>OR<br>Any person with inexplicable bleeding/ haemorrhaging
Confirmed case	Any suspected case with laboratory confirmation (PCR is the key diagnostic tool used here).	
Deaths	Cases that die either as suspected or confirmed cases.	
Alert threshold	A single suspected case of Lassa fever.	
Outbreak threshold	A single confirmed case of Lassa fever. Clinicians should have a high index of suspicion when managing febrile illnesses; especially cases with:	a. A history of non-response to antimalarials or antibiotics.<br>b. A compatible history of travel to an endemic area or an area with an ongoing outbreak, contact with a confirmed case of Lassa fever, negative thick blood film for malaria parasite are suggestive.<br>c. Signs of haemorrhage and shock which is strongly suggestive, but these signs often appear late in the illness.

Supplementary Table 2: Metrics of model fit for spatial Lassa fever occurrence and incidence models. Tables show the comparison of model information criteria (Deviance Information Criterion DIC and Watanabe-Akaike Information Criterion WAIC) between baseline random-effects only models, and models with socio-ecological and climate covariates (model results shown in Figure 3). Occurrence models were specified with a binomial likelihood (modelling annual presence-absence of Lassa fever) and incidence models were specified with a zero-inflated Poisson likelihood and an offset for log population (modelling annual incidence of Lassa fever).

Response	Model	DIC	WAIC	Socio-ecological covariates
Occurrence	Baseline	1708.51	1591.49	None
Occurrence	Socio-ecological	1547.46	1463.67	Total annual precipitation (nonlinear), Mean annual temperature, Urban cover, Agriculture cover, Proportion of the population in poverty
Incidence	Baseline	3107.36	3311.04	None
Incidence	Socio-ecological	2912.20	3062.76	Total annual precipitation (nonlinear), Urban cover, Agriculture cover, Proportion of the population in poverty, Travel time to Lassa diagnostic lab

Supplementary Table 3: Parameter estimates from the spatial models of Lassa fever occurrence and incidence. Tables show posterior marginal fixed effects parameter and hyperparameter estimates (median and 95% credible interval) from models of annual Lassa fever occurrence and incidence at LGA-level (occurrence n=774 LGAs over 4 years) and at aggregated district level (n=130 districts over 4 years). Occurrence was modelled using a binomial error distribution so estimates are on the log-odds scale, and incidence with zero-inflated Poisson (log link) so estimates are on the natural logarithmic scale. All fixed effect covariates were scaled (mean 0, sd 1) prior to model fitting so parameter estimates reflect the effect of a change in 1 scaled unit (standard deviation) of the covariate on the response variable (Methods).

Model	Response	Type	Name	Median	CI_0.025	CI_0.975
LGA	Occurrence	fixed effect	Intercept	-4.093	-4.684	-3.588
LGA	Occurrence	fixed effect	Temperature (mean annual)	-0.322	-0.530	-0.113
LGA	Occurrence	fixed effect	Built up land proportion	0.910	0.646	1.190
LGA	Occurrence	fixed effect	Agricultural land proportion	0.461	0.212	0.724
LGA	Occurrence	fixed effect	Poverty prevalence	0.481	0.089	0.888
LGA	Occurrence	hyperparam	Precision for LGA (bym2)	0.365	0.250	0.539
LGA	Occurrence	hyperparam	Phi for LGA (bym2)	0.987	0.933	0.999
LGA	Occurrence	hyperparam	Precision for mean annual precipitation (random walk)	5.411	2.090	14.532
LGA	Incidence	fixed effect	Intercept	-16.177	-16.679	-15.721
LGA	Incidence	fixed effect	Built up land proportion	0.807	0.533	1.085
LGA	Incidence	fixed effect	Agricultural land proportion	0.381	0.120	0.649
LGA	Incidence	fixed effect	Poverty prevalence	0.971	0.523	1.431
LGA	Incidence	fixed effect	Distance from Lassa lab	-1.201	-1.710	-0.721
LGA	Incidence	hyperparam	Zero probability parameter z (zero-inflated Poisson)	0.012	0.001	0.067
LGA	Incidence	hyperparam	Precision for LGA (bym2)	0.179	0.144	0.212
LGA	Incidence	hyperparam	Phi for LGA (bym2)	0.968	0.894	0.995
LGA	Incidence	hyperparam	Precision for mean annual precipitation (random walk)	21.303	3.704	306.753
District	Occurrence	fixed effect	Intercept	-1.303	-1.794	-0.953
District	Occurrence	fixed effect	Temperature (mean annual)	-0.595	-0.926	-0.292
District	Occurrence	fixed effect	Built up land proportion	0.554	0.266	0.887
District	Occurrence	fixed effect	Agricultural land proportion	0.441	0.130	0.769
District	Occurrence	fixed effect	Poverty prevalence	-0.289	-0.715	0.122
District	Occurrence	hyperparam	Precision for District (bym2)	1.645	0.480	7.730
District	Occurrence	hyperparam	Phi for District (bym2)	0.795	0.184	0.986
District	Occurrence	hyperparam	Precision for mean annual precipitation (random walk)	18.235	4.534	96.849
District	Incidence	fixed effect	Intercept	-16.033	-16.517	-15.615
District	Incidence	fixed effect	Built up land proportion	0.204	-0.141	0.564
District	Incidence	fixed effect	Agricultural land proportion	0.120	-0.263	0.508
District	Incidence	fixed effect	Poverty prevalence	0.389	-0.191	0.993
District	Incidence	fixed effect	Distance from Lassa lab	-1.454	-2.065	-0.863
District	Incidence	hyperparam	Zero probability parameter z (zero-inflated Poisson)	0.010	0.001	0.068
District	Incidence	hyperparam	Precision for District (bym2)	0.172	0.126	0.221
District	Incidence	hyperparam	Phi for District (bym2)	0.906	0.711	0.986
District	Incidence	hyperparam	Precision for mean annual precipitation (random walk)	217.339	5.043	58924.366

Supplementary Table 4: Lassa-fever endemic states included in the temporal models. The table shows the name and state of each spatially aggregated district included in temporal incidence models, the local government authorities (LGAs) within each district and the total number of confirmed cases detected across all years of surveillance. Cases from 2012-2016 are from the Weekly Epidemiological Reports regime, and from 2017-2019 are from the NCDC Situation Reports surveillance regime.

State	Region	Cases total (2012-2019)	Cases 2017-2019
Edo	South	1333	883
Ondo	South	723	601
Ebonyi	South	244	203
Plateau	North	114	71
Taraba	North	144	84
Bauchi	North	143	93

Supplementary Table 5: Data sources for all covariates included in analyses. The table includes the sources and rationale (hypothesis) for inclusion of covariates in spatial and spatiotemporal models of Lassa fever incidence across Nigeria. Modelling is described in full in Methods.

Covariate	Type	Units	Time period	Model	Rationale	Data source, methods	Potential sources of error/misspecification
Mean travel time from laboratory with LF diagnostic capacity	Con	Km	Annual	Spat., Temp.	Lassa fever detections were historically geographically biased towards areas near to diagnostic laboratories (e.g. ISTH; Gibb et al 2017)	Derived from diagnostic laboratory locations (via NCDC) and travel time estimated using friction surface (https://malariaatlas.org/research-project/accessibility-to-cities/).	Distance does not necessarily equate to access and there are different capacities for each laboratory. Health-seeking behaviour will drive small-scale patterns.
Mean travel time to nearest hospital	Con	Km	2015	Spat.	Proxy for public access to larger/regional healthcare facilities with links to state-level surveillance infrastructure	Estimated as distances from geolocated hospitals in Maina *et al*., 2019[47] and travel time estimated using friction surface (https://malariaatlas.org/research-project/accessibility-to-cities/).	Distance does not necessarily linearly equate to access. Health-seeking behaviour will drive small-scale patterns.
State ID	Cat	n/a	n/a	Spat.	State-level differences in LF surveillance and awareness may influence sensitivity of reporting	n/a	Surveillance and awareness will likely not conform to exact boundaries and spatial scale may be moderately mismatched
Air temperature (annual mean and seasonality)	Con	ºC	2011-19, annual	Spat.	Temperature may affect the environmental suitability for *M. natalensis* and/or viral transmission (e.g. through affecting persistence in the environment).	Derived from daily temperature averages from NOAA CPC (https://www.esrl.noaa.gov/psd/data/gridded/data.cpc.globaltemp.html)	Interpolated estimates across state and time will differ slightly from exact values. It is a broad proxy, as food growth, env. stress and viral persistence will all vary with microclimate and host behavioural variation
Precipitation (mean wettest month, mean driest month)	Con	mm	2011-19, annual	Spat.	Evidence of links between *M. natalensis* population ecology and rainfall seasonality, and past evidence that human LF is linked to areas of high rainfall[4]	Derived from daily Africa precipitation rasters from CHIRPS (Climate Hazards Infrared Precipitation with Stations)[42] (https://www.chc.ucsb.edu/data/chirps)	Interpolated estimates across state and time will differ slightly from exact values. It is a broad proxy, as food growth, env. stress and viral persistence will all vary with microclimate and host behavioural variation
Precipitation annual seasonality (coefficient of variation)	Con	mm	2011-19, monthly	Spat.	Evidence of links between *M. natalensis* population ecology and rainfall seasonality, and past evidence that human LF is linked to areas of high rainfall[4]	Derived from daily Africa precipitation rasters from CHIRPS (Climate Hazards Infrared Precipitation with Stations)[42] (https://www.chc.ucsb.edu/data/chirps)	Interpolated estimates across state and time will differ slightly from exact values. It is a broad proxy, as food growth, env. stress and viral persistence will all vary with microclimate

							and host behavioural variation
Mean precipitation in 60-day window (starting 0, 30, 60, 90, 120 day lag prior to reporting week)	Con	mm	2011-20, weekly	Temp.	Evidence that *M. natalensis* population densities and patterns of human exposure may be linked to rainfall patterns[4]	Derived from daily Africa precipitation rasters from CHIRPS[42] (https://www.chc.ucsb.edu/data/chirps)	Interpolated estimates across state and time will differ slightly from exact values. It is a broad proxy, as food growth, env. stress and viral persistence will all vary with microclimate and host behavioural variation
Standardised Precipitation Index in 60-day window (starting 0, 30, 60, 90, 120 day lag prior to reporting week)	Con	Scaled units	2011-20, weekly	Temp.	Evidence that *M. natalensis* population densities and patterns of human exposure may be linked to rainfall patterns[4]. SPEI is a measure of drought or wetness relative to the historical trends for the same month of the year.	Calculated from long-term Africa precipitation time-series (1981-2020) from CHIRPS[42] using R package 'spei' (https://www.chc.ucsb.edu/data/chirps)	Interpolated estimates across state and time will differ slightly from exact values. It is a broad proxy, as food growth, env. stress and viral persistence will all vary with microclimate and host behavioural variation
Mean, min and max air temperature in 60-day window (starting 0, 30, 60, 90, 120 day lag prior to reporting week)	Con	ºC	2011-20, weekly	Temp.	*M. natalensis* population densities, viral persistence and patterns of human exposure may be linked to climatic patterns[4]	Derived from daily Tmin and Tmax temperature layers from NOAA CPC (https://www.esrl.noaa.gov/psd/data/gridded/data.cpc.globaltemp.html)	Interpolated estimates across state and time will differ slightly from exact values. It is a broad proxy, as food growth, env. stress and viral persistence will all vary with microclimate and host behavioural variation
Mean EVI in 60-day window (starting 0, 30, 60, 90, 120 day lag prior to reporting week)	Con	EVI	2011-20, weekly	Temp.	Evidence that *M. natalensis* population densities and patterns of human exposure may be linked to climatically-driven vegetation dynamics (i.e. due to seasonal resource availability)[4]	Derived from processed 16-day EVI MOD13A2 layers from NASA (https://lpdaac.usgs.gov/products/mod13a2v006)	Interpolated estimates across state and time will differ slightly from exact values. It is a broad proxy, as food growth, env. stress and viral persistence will all vary with microclimate and host behavioural variation
Urban land cover	Con	Prop. area	2015	Spat.	May impact both *M. natalensis* densities (which are often lower in highly urbanised environments) and human access to healthcare facilities (i.e. detection probability)	ESA-CCI Land Cover 2015 300m raster (data for a single year as relatively low change in proportion cover during surveillance period) (https://www.esa-landcover-cci.org)	Gross land cover categorisation will not fully capture the high variation and difference between difference habitat types. The quality of the habitats, their fragmentation and the geographical differences in such habitats will also likely be important.

Agricultural land cover	Con	Prop. area	2015	Spat.	*M. natalensis* densities often observed to be highest in agricultural settings[9,30] and cropping and crop preparation practices are a hypothesised driver of human-rodent contact[4]	ESA-CCI Land Cover 2015 (data for a single year as relatively low change in proportion cover during surveillance period) (https://www.esa-landcover-cci.org/)	Gross land cover categorisation will not fully capture the high variation and difference between difference habitat types. The quality of the habitats, their fragmentation and the geographical differences in such habitats will also likely be important.
Total human population (log transformed)	Con	No. people	2012-20, annual	Spat., Temp.	Controlling for effects of increased human population on potential for LASV exposure.	WorldPop (https://www.worldpop.org/geodata/summary?id=1437)	Does not account for exact proportion of people that at-risk and how this varies over space and time. More accurate might be density of agricultural workers for instance.
Proportion of LGA population living in poverty (<$1.25 per day)	Con	Prop. area	2010	Spat.	Evidence that human LASV exposure may often be linked to ability to store food in rodent-proof containers and ability to rodent-proof housing[10,54]	WorldPop (https://www.worldpop.org/geodata/summary?id=1267)	Poverty is a broad proxy for ability for people to prevent or react to disease risk. Differences e.g. in awareness, personal choice and beliefs, and personal circumstances will influence actions.
Proportion of population in locales with improved housing	Con	Prop. area	2015	Spat.	Evidence that human LASV exposure may be linked to ability to rodent-proof housing[54]	Derived from Malaria Atlas Project modelled data on prevalence of improved housing[45] ($5km^2$ resolution) and human population layers (WorldPop)	This index intends to capture the ability of rodents to exploit food in houses but the actual design and use of house will have strong impact on how many host individuals are able to invade.
