# Supplementary material for: Geographical drivers and climate-linked dynamics of Lassa fever in Nigeria
Source: Nat Commun. 2021 Oct 1;12:5759. doi: 10.1038/s41467-021-25910-y (PMC8486829; doi:10.1038/s41467-021-25910-y)
Supplement: Supplementary file 3 — Reporting Summary [file 41467_2021_25910_MOESM3_ESM.pdf]

nature portfolio

Corresponding author(s): David Redding

Last updated by author(s): 01/09/2021

Reporting Summary

Nature Portfolio wishes to improve the reproducibility of the work that we publish. This form provides structure for consistency and transparency in reporting. For further information on Nature Portfolio policies, see our Editorial Policies and the Editorial Policy Checklist.

Statistics

For all statistical analyses, confirm that the following items are present in the figure legend, table legend, main text, or Methods section.

n/a	Confirmed	
☐	☒	The exact sample size (*n*) for each experimental group/condition, given as a discrete number and unit of measurement
☐	☒	A statement on whether measurements were taken from distinct samples or whether the same sample was measured repeatedly
☒	☐	The statistical test(s) used AND whether they are one- or two-sided<br>*Only common tests should be described solely by name; describe more complex techniques in the Methods section.*
☐	☒	A description of all covariates tested
☐	☒	A description of any assumptions or corrections, such as tests of normality and adjustment for multiple comparisons
☐	☒	A full description of the statistical parameters including central tendency (e.g. means) or other basic estimates (e.g. regression coefficient) AND variation (e.g. standard deviation) or associated estimates of uncertainty (e.g. confidence intervals)
☒	☐	For null hypothesis testing, the test statistic (e.g. *F*, *t*, *r*) with confidence intervals, effect sizes, degrees of freedom and *P* value noted<br>*Give P values as exact values whenever suitable.*
☐	☒	For Bayesian analysis, information on the choice of priors and Markov chain Monte Carlo settings
☐	☒	For hierarchical and complex designs, identification of the appropriate level for tests and full reporting of outcomes
☒	☐	Estimates of effect sizes (e.g. Cohen's *d*, Pearson's *r*), indicating how they were calculated

Our web collection on statistics for biologists contains articles on many of the points above.

Software and code

Policy information about availability of computer code

Data collection	Data were collected and stored in Microsoft Excel 2019
Data analysis	Date were analysed using R v.3.4.1. Analyses were undertaken using R packages R-INLA v.20.03.1737, raster v.3.4.1338 and velox v0.2.039. All code used for these analyses are provided at the accompanying repository DOI: 10.6084/m9.figshare.9777656.

For manuscripts utilizing custom algorithms or software that are central to the research but not yet described in published literature, software must be made available to editors and reviewers. We strongly encourage code deposition in a community repository (e.g. GitHub). See the Nature Portfolio guidelines for submitting code & software for further information.

Data

Policy information about availability of data

All manuscripts must include a data availability statement. This statement should provide the following information, where applicable:

- Accession codes, unique identifiers, or web links for publicly available datasets
- A description of any restrictions on data availability
- For clinical datasets or third party data, please ensure that the statement adheres to our policy

The Lassa Fever case data collected in this study have been deposited in the figshare database under accession code m9.figshare.9777656.v1 [https://doi.org/10.6084/m9.figshare.9777656.v1]. These data are openly available under the CC BY 4.0 license and can be obtained from the link above. Further data generated in this study are provided in the Supplementary Information.

Field-specific reporting

Please select the one below that is the best fit for your research. If you are not sure, read the appropriate sections before making your selection.

☒ Life sciences ☐ Behavioural & social sciences ☐ Ecological, evolutionary & environmental sciences

For a reference copy of the document with all sections, see nature.com/documents/nr-reporting-summary-flat.pdf

Life sciences study design

All studies must disclose on these points even when the disclosure is negative.

Sample size	The data set we had represented all available data points for Lassa cases in Nigeria from 2012-2019. We subsetted this complete data set for two analyses: For the spatial models we used annual confirmed case counts for all LGAs across the last 4 years of surveillance (2016 to 2019) since these years followed the establishment of updated systematic surveillance protocols and the associated geographical expansion of suspected case reports (Figure 2), and so are likely to more fully represent the true underlying distribution of LF across Nigeria (n=3096). For the temporal models, we used LF incidence (weekly case counts) across the full duration of surveillance (2012 to 2019) but only included the high incidence states to reduce zero-inflation (Edo, Ondo, Ebonyi, Bauchi, Plateau and Taraba states), which in total account for 87% of total confirmed cases since 2012 (n=2820). Given this is a Bayesian analysis the relationships described in the results are only those for which there are sufficient data to infer them, thus the sample size was large enough to uncover the relationships that we reported.
Data exclusions	No data points were excluded from the analysis overall.
Replication	To investigate additional effects of environmental conditions on interannual LF outbreak dynamics and evaluate scope for forecasting, we conducted out-of-sample (OOS) based model selection for linear and nonlinear effects of climate covariates. We considered candidate models for all lagged combinations of all 4 covariates, and identified the model that minimised out-of-sample (OOS) predictive error (measured as root mean square error, RMSE) on sequential 6-month holdout windows across the study period.
Randomization	We used previously collected data on disease cases - we could not randomize anything about the data collection it was simply all known cases for the disease we are examining. See above for randomization in the cross-validation procedure.
Blinding	Blinding was not appropriate as the data were historically reported disease cases - this was not an intervention experiment - all the data were collected in the past so we had no say in how they were collected.

Reporting for specific materials, systems and methods

We require information from authors about some types of materials, experimental systems and methods used in many studies. Here, indicate whether each material, system or method listed is relevant to your study. If you are not sure if a list item applies to your research, read the appropriate section before selecting a response.

Materials & experimental systems

n/a	Involved in the study	
☒	☐	Antibodies
☒	☐	Eukaryotic cell lines
☒	☐	Palaeontology and archaeology
☒	☐	Animals and other organisms
☒	☐	Human research participants
☒	☐	Clinical data
☒	☐	Dual use research of concern

Methods

n/a	Involved in the study	
☒	☐	ChIP-seq
☒	☐	Flow cytometry
☒	☐	MRI-based neuroimaging
